# Real-Time Location Tracking of Multiple Construction Laborers

**DOI:** 10.3390/s16111869

**Published:** 2016-11-06

**Authors:** Jin-Sun Lim, Ki-Il Song, Hang-Lo Lee

**Affiliations:** 1 R&D Center, SamwooIMC CO., LTD, Seoul 05660, Korea; coreplay@hanmail.net; 2 Department of Civil Engineering, Inha University, Incheon 22212, Korea; hanglolee@inha.edu

**Keywords:** construction site, RTL system, radio signal strength indicator, accelerometer

## Abstract

A real-time location (RTL) system was developed to improve safety for multiple laborers in confined construction sites. The RTL system can monitor the location and movement of multiple laborers in real time. A portable RTL system with a low-battery mode was developed to accommodate various constraints in the construction site. A conventional RTL system that uses radio signal strength indicators (RSSIs) has high error, so an accelerometer with Bluetooth Low Energy (BLE) was added, and a calculation process is suggested. Field tests were performed for validation in underground construction and bridge overlay sites. The results show that the accelerometer and BLE can be used as effective sensors to detect the movement of laborers. When the sensor is fixed, the average error ranges 0.2–0.22 m, and when the sensor is moving, the average error ranges 0.1–0.47 m.

## 1. Introduction

An effective monitoring system is needed for construction management and safety control. The safety of laborers and construction vehicles is essential in confined construction sites such as in underground spaces. Innovative monitoring sensors and monitoring systems have been introduced for construction materials, machines, and laborers [1,2,3,4,5,6]. Among the various sensing techniques, real-time location (RTL) systems are widely known as a monitoring technique for the management of machines and laborers using radio frequency identification (RFID) tags. Lee et al. [7] and Costin et al. [8] introduced RFID for monitoring and management of construction material at construction sites.

Wireless positioning techniques using global positioning system (GPS), which is only available in open spaces, cannot be implemented in confined construction sites. Thus, an alternative RTL system is needed. Park et al. [9] carried out a feasibility analysis of an RTL system in various construction sites. Kim et al. [5] established an RTL system using a wireless communication technique based on Wi-Fi in a tunnel construction site for the safety monitoring of laborers with a single tag. Lee et al. [10] applied an RTL system based on the chirp spread spectrum (CSS) technique within and outside of a building construction site.

Moreover, labor consumption measurements at construction sites is proposed by Jiang et al. [11] using GPS, geographic information system (GIS) and wideband code division multiple access networks. Khoury et al. [12] combined a vision-based tracking system with a foot-mounted inertia measurement unit (IMU) to monitor laborers at construction sites. Ray and Teizer [13] performed posture estimation of construction laborers and classification using a real-time range camera. Most previous studies used a single sensor. Moreover, a system for monitoring multiple laborers has not been introduced previously.

In this study, an RTL system that can monitor the movement pattern and location of multiple laborers was developed. To improve the location tracking algorithm used in current RTL systems, an accelerometer that supports Bluetooth Low Energy (BLE) was added, and an effective search algorithm is introduced. The system was implemented in construction sites for validation. The results show that the system can be used as an effective tool for the management and safety of laborers at a construction site. 

## 2. Development of the RTL System

### 2.1. Structure of the RTL System

**Figure 1:**
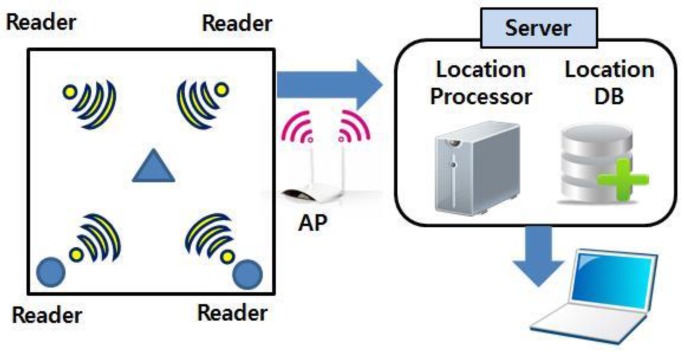
Structure of RTL system for monitoring multiple sensors.

As shown in Figure 1, the RTL system is composed of tags that periodically transfer information such as the ID and location to a reader (a fixed node). After reception, the fixed node transmits a signal to a server. The information is collected from each tag by the server, processed, stored, and managed in a database, and the results are sent to various devices [14].

### 2.2. Wireless Network System

**Figure 2:**
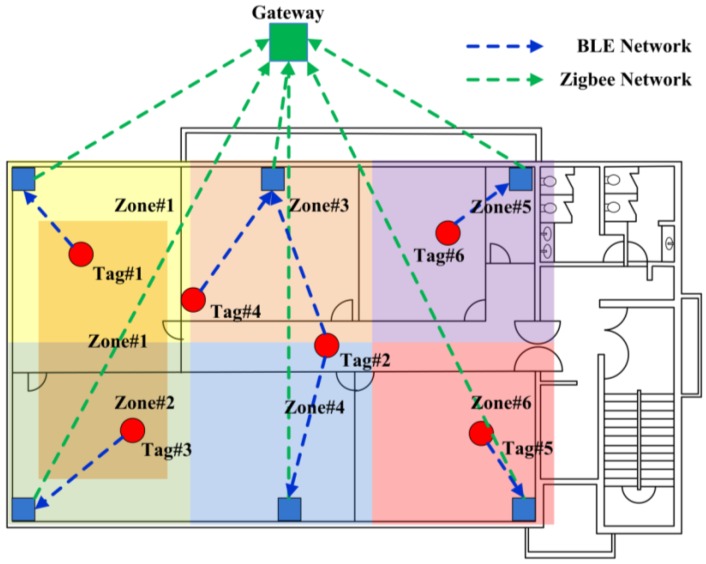
Configuration of network in a building construction project.

Wireless network methods are used in the RTL system, and the configuration of a network is shown in Figure 2. The most representative wireless methods are Wi-Fi, Bluetooth, ZigBee, and Ultra-Wide Band (UWB). Table 1 compares some of these technologies [15].

In the case of Wi-Fi, developing a system with a battery is not easy because the maximum transmission distance of Wi-Fi is very large, and the power consumption is very high. On the other hand, Bluetooth has the shortest transmission distance, and its transmission power consumption level is low. As for ZigBee, although its transmission rate is very low and limited when transferring a large amount of information, it has excellent characteristics in terms of energy consumption and grid-based network construction. UWB has the fastest data transmission rate but a short transmission distance, and it also needs expensive equipment.

Without consideration of cost, it is possible to develop an outstanding RTL communication system using any of these methods. However, in this study, Bluetooth was selected in order to minimize the power for the tags and fixed nodes and to set up a portable system. Furthermore, Bluetooth easily sends various sensor signals integrally. ZigBee is flexible and expandable and would be favorable for location information since the data size is relatively small compared to audio and video files [16].

## 3. Determination of Location

### 3.1. Determination of Location with RSSI

The received signal strength indication (RSSI) is an index of signal strength and a major variable in the distance measurement algorithm based on Bluetooth. Theoretically, the strength of a radio signal is inversely proportional to the square of the distance, and the RSSI is arbitrarily set in proportion to the logarithm of the strength using the Firris equation [17,18]:

(1) $$L=20\;\log_{10}\left(\frac{4\pi d}{\lambda }\right)\left(\mathrm{dB}\right)$$

where *L* is the strength of the radio signal, λ is the wavelength, and *d* is the distance. If *d* is the distance between two points, Equation (1) can then be rewritten as:

(2) $$d=\frac{\lambda }{4\pi }\cdot 10^{\frac{L}{20}}=\frac{c}{4\pi f}\cdot 10^{\frac{L}{20}}$$

where *c* is the velocity of wave propagation, and *f* is the frequency.

**Figure 3:**
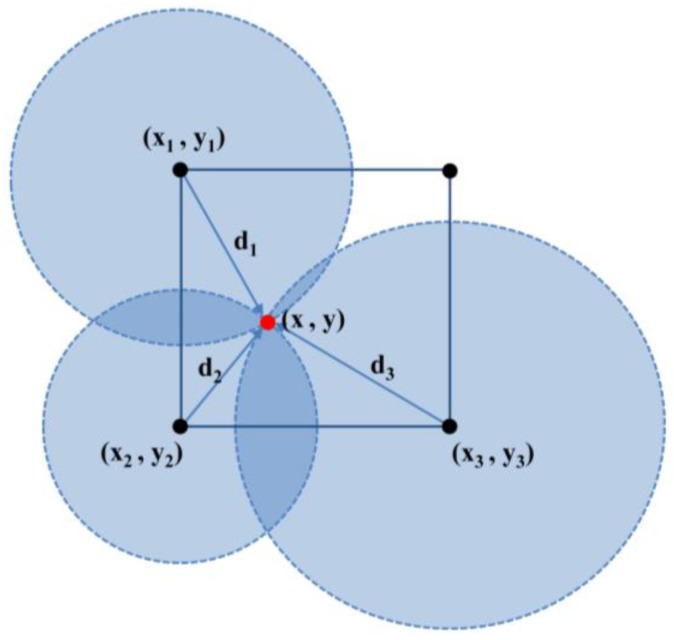
Determination of location using triangular survey in RTL system.

When the RSSI is transmitted between three fixed nodes as shown in Figure 3, the location can be determined by using the triangular survey method:

(3) $${\left(x-x_{1}\right)}^{2}+{\left(y-y_{1}\right)}^{2}=d_{1}^{\;2}\;{\left(x-x_{2}\right)}^{2}+{\left(y-y_{2}\right)}^{2}=d_{2}^{\;2}\;{\left(x-x_{3}\right)}^{2}+{\left(y-y_{3}\right)}^{2}=d_{3}^{\;2}$$

However, due to the direction and angle of the radio antenna, obstructions between transmission devices, and interference with the radio antenna, large error and sensitivity in the position measurement can occur. The error of the actual received signal is included in Equation (4):

(4) $${\left(x-x_{1}\right)}^{2}+{\left(y-y_{1}\right)}^{2}=d_{1}^{\;2}-{\mathrm{er}}_{1}\;{\left(x-x_{2}\right)}^{2}+{\left(y-y_{2}\right)}^{2}=d_{2}^{\;2}-{\mathrm{er}}_{2}\;{\left(x-x_{3}\right)}^{2}+{\left(y-y_{3}\right)}^{2}=d_{3}^{\;2}-{\mathrm{er}}_{3}$$

where er_1_, er_2_, and er_3_ are the errors of the signal transmission. Since the error of a moving tag can accumulate, the results may not be reliable. For this reason, in previous research on the determination of the position using the RSSI, the problems were overcome by applying algorithms to estimate the position [19,20].

In this study, in order to record the position tracking data continuously, the least square method based on a Kalman filter was used to minimize the measurement error. The Kalman filter is expressed by the mean and variance of the noise, assuming a normal distribution. The Gaussian distribution can be expressed as Equation (5) [21]:

(5) $$x_{k}=A\cdot x_{k\;-\;1}+B\cdot u_{k}+w_{k\;-\;1}\;z_{k}=H\cdot x_{k}+v_{k}\;p\left(w\right)\sim N\left(0,Q\right)\;p\left(v\right)\sim N\left(0,R\right)$$

where x_k_ is the state vector at time k, x_k − 1_ is the state vector at time k − 1, u_k_ is the input variable from the user, w_k − 1_ is the noise at k − 1, v_k_ is the measured noise at k, z_k_ is the position vector that includes the noise, A is the state transition matrix based on the previous time in the current time, B is the input matrix, H is a matrix related to the measured time, and Q and R are covariance matrixes. If there are three signal readers, a Kalman filter can be applied after one or more data are accumulated for at least nine time steps in the case of triangular survey.

### 3.2. Determination of Position with Accelerometer

In order to overcome the inaccurate summation of the estimated positions of the RSSI, a three-axis accelerometer and Bluetooth Low Energy (BLE) are adopted. The accelerometer measures the acceleration in the three directions of the sensor in time intervals, from which the tag’s moving distance, gradient, and moving direction can be determined [22]. In the accelerometer graph, two of the three axes are in the direction of gravity and the vertical direction. The remaining axis is in the direction of gravity with a measured acceleration of 9.8 m/s. If the direction of the acceleration does not match with the direction of gravity, the output will be changed. The location can be determined by the accelerometer, but it can cause an error. It is not easy to remove the gravity error. When combined with GPS or an RTL construction system, the acceleration due to gravity and pure motion acceleration can be separated from the total acceleration, through which the estimation of the error of the moving distance can be reduced [22].

Further, a_x_, a_y_, and a_z_ are the accelerations in the X, Y, and Z directions, assuming the Z-axis indicates the direction of gravity. Using the angle of rotation θ_y_ about the Y-axis, and the acceleration with the gravity, effect a_xz_ removed can be obtained from Equations (6) and (7):

(6) $$\theta_{y}=90-\cos^{-1}\left(\frac{a_{x}}{1G}\right)$$

(7) $$a_{\mathrm{xz}}=\sqrt{{\left(a_{x}-1G\cdot {\sin\theta }_{y}\right)}^{2}+{\left(a_{z}-1G\cdot {\sin\theta }_{y}\right)}^{2}}$$

The angle between acceleration a_xz_ and the horizontal direction is represented by Equation (8), and acceleration a_Hx_, which considers gravity, can be expressed as Equation (9).

(8) $$\theta_{\mathrm{xz}}=\theta_{y}-\cos^{-1}\left(\frac{a_{x}-G\cdot {\sin\theta }_{y}}{a_{\mathrm{xz}}}\right)$$

(9) $$a_{\mathrm{Hx}}=\cos\theta_{xz}\cdot a_{\mathrm{xz}}$$

Similarly, θ_x_, a_yz_, θ_yz_, and a_Hy_ can be found with respect to the X-axis. The pure motion accelerations a_mHx_ and a_mHy_ in the Hx and Hy directions can be expressed as Equations (10) and (11), respectively.

(10) $$a_{\mathrm{mHx}}=a_{x}\theta_{y}-a_{\mathrm{Hx}}$$

(11) $$a_{\mathrm{mHx}}=a_{x}\theta_{y}-a_{\mathrm{Hx}}$$

Using a double time integral, the moving distances S_x_ and S_y_ in the Hx and Hy directions in a unit of time can be estimated, and the moving distance in all horizontal directions can be represented using Equation (12).

(12) $$s=\sqrt{S_{x}^{\;2}+S_{y}^{\;2}}$$

The direction vector at n − 1 is S_n − 1_, and the direction vector in moving n time is S_n_. The angle $\phi_{n}$ of the moving direction of a moving tag in a unit of time can be represented as Equation (13):

(13) $$\phi_{n}=\phi_{n\;-\;1}+\cos^{-1}\frac{S_{n}\cdot S_{n\;-\;1}}{∥S_{n}∥∥S_{n\;-\;1}∥}$$

### 3.3. Tracking for Multiple Sensors

The BLE tag transmits signals at an average of 20 Hz. When a fixed node reads the signal transmitted from about 20 tags, there is a delay of two seconds. Using the triangular survey with the RSSI method, the minimum scan interval is about two seconds. Thus, the location information can be updated every two seconds. Even though there is no error in the position information when a tag moves at 0.5 m/s, 1 m of error is inevitable in real-time measurement. For long-term management of laborers, two seconds of delay for data transmission is not significant.

To improve the performance of the RTL system, an accelerometer is combined with the RSSI for a single fixed node. Four fixed nodes scan moving nodes sequentially, and a time interval of 0.5 s is adopted. In order to minimize the loss of data induced by the error of the RSSI signal, the maximum allowable movement distance (MAMD) is introduced to the position determination process. MAMD is the maximum distance that a laborer can move within a unit of time in the construction site. Since MAMD is affected by the unit of time, the errors involved with distance measurement can be reduced as the required time for scanning decreases. The moving speed of a laborer is assumed to be less than 0.5 m per second, and an MAMAD of 0.25 m is determined for an underground construction site.

**Figure 4:**
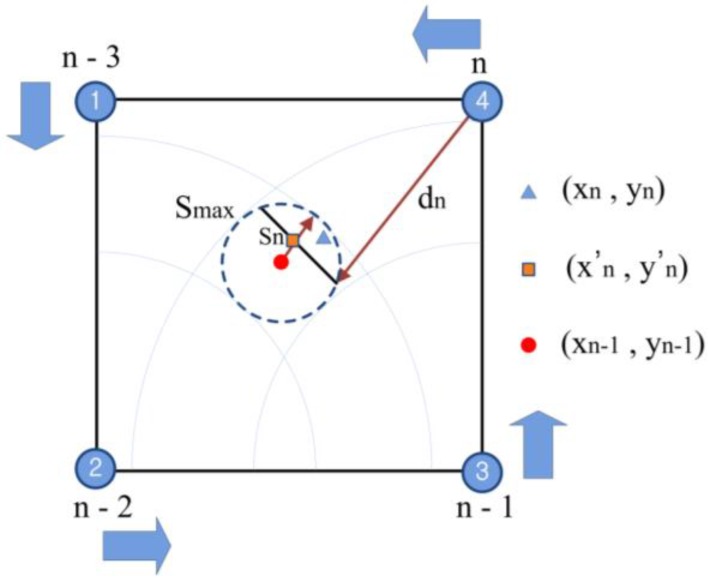
Searching and tracking method in RTL system.

Figure 4 shows the concept of the location tracing. The location of the moving tag is initialized, the RSSI signal from the four fixed nodes is detected, and the distance *d_n_* is estimated.

**Figure 5:**
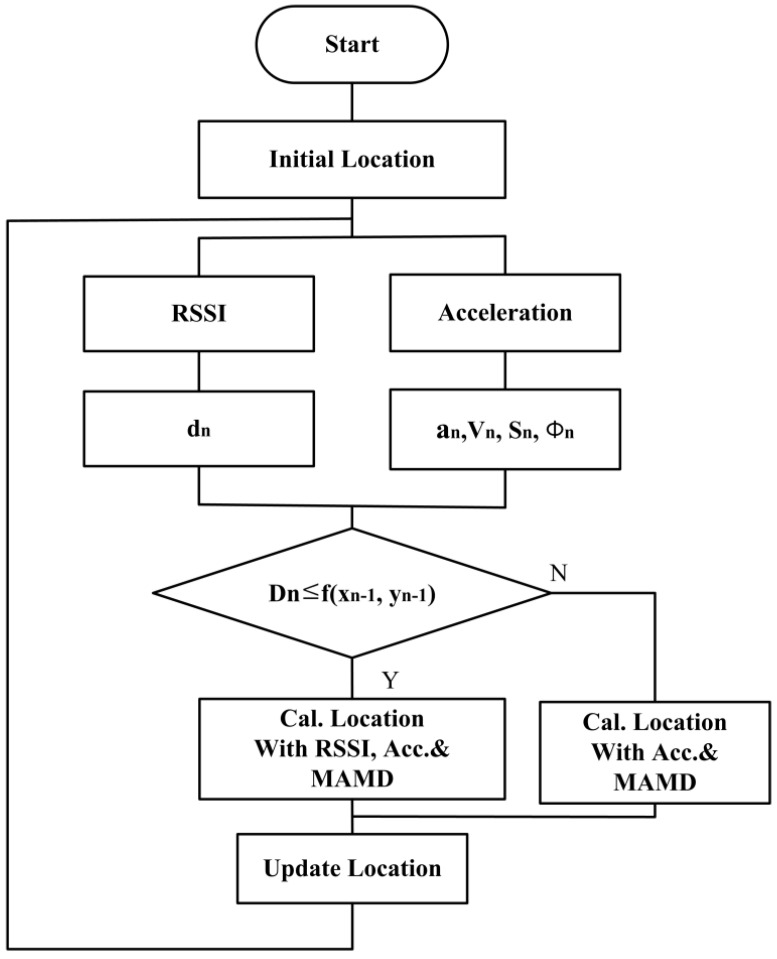
Flow of positioning with RSSI and acceleration.

Assuming that the location of the red point at n − 1 moves to the location of the blue triangle at *n*, we can compare the circle with the radius equal to MAMD and the center $\left(x_{n\;-\;1},y_{n\;-\;1}\right)$ to the semicircle obtained with the RSSI from the location of the four fixed nodes at *n*. When they meet each other, as shown in Figure 4, the location of the square, $\left(x_{n}^{\prime }\;,{\;y}_{n}^{\prime }\right)$, and the optimal solution can be determined. When they do not meet each other, the location is only determined by the accelerometer, and the database is updated. Figure 5 shows the flow of the positioning algorithm.

## 4. Application of RTL System

### 4.1. Evaluation on the Applicability of the RTL System in an Underground Construction Site

**Figure 6:**
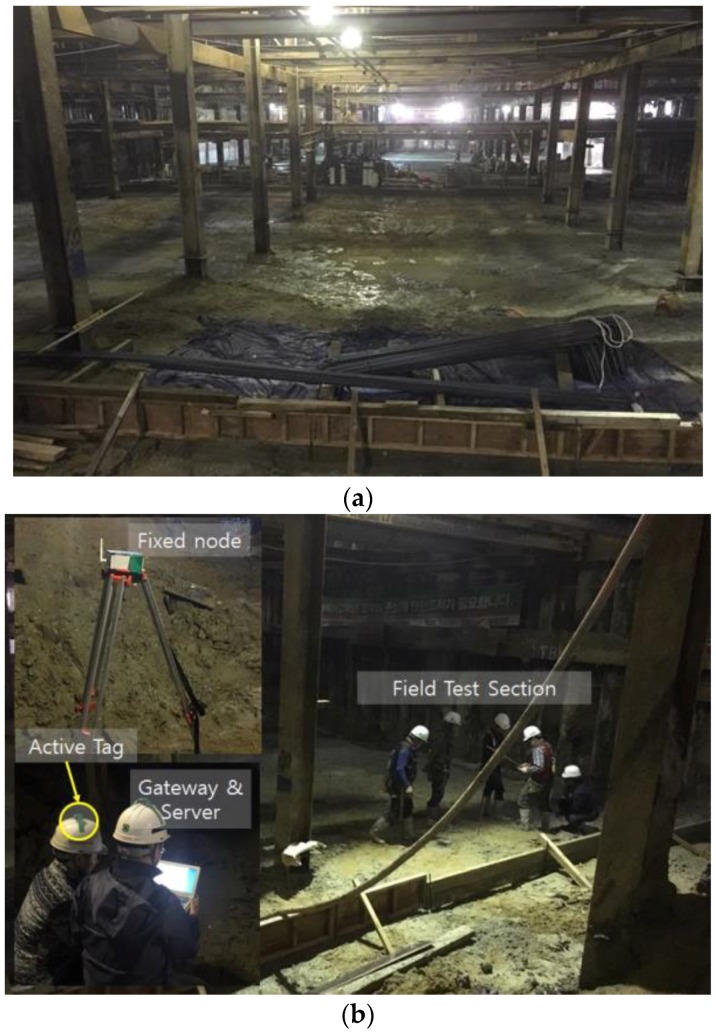
Experimental test in underground construction site: (**a**) Underground construction site; (**b**) Components of RTL system.

A site test is conducted to evaluate the performance of the system at an underground construction site in Incheon, South Korea. Figure 6a shows a picture of the site, and Figure 6b shows the test equipment and components. The test evaluates the performance of the tracking technique in a closed space. First, the function and error of the proposed methods are assessed. Then, a tag that is moving through a glide path and position tracking data are evaluated.

#### 4.1.1. Location Tracking of Fixed Tag

**Figure 7:**
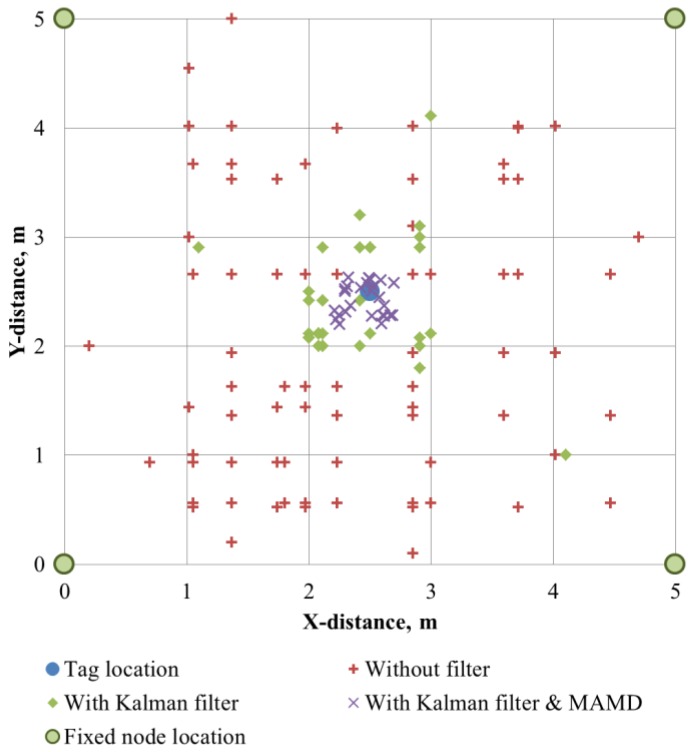
Detection of location for fixed tag in underground construction site.

To evaluate the location tracking capability of the fixed tags, four fixed nodes were installed at four corners in an area of 5 m × 5 m, and a tag attached to a helmet was placed at the center of the testing area. The interval of the fixed node was determined as 5 m in consideration of the performance of the BLE. Figure 7 shows the result of the location detection for 1 min.

Table 2 shows the errors obtained from 10 instances of identical experimental tests. The error can be obtained from Equation (14):

(14) $$\mathrm{error}=\sqrt{{\left(x_{e}-x_{f}\right)}^{2}+{\left(y_{e}-y_{f}\right)}^{2}}$$

where x_e_ and y_e_ are the estimated tag position, and x_f_ and y_f_ are the fixed tag position. 

The average error was 1.56–1.82 m, and a maximum error of 2.92–4.88 m was obtained when only triangular surveying was conducted with the RSSI. Even though the location of the tag was fixed, the error was bigger than expected. This indicates that the location tracking and traveling path prediction using only triangular surveying and RSSI are not feasible. When the Kalman filter was implemented, the accuracy of the detection was improved significantly. As the data accumulated, the range of the error decreased, and the average distance error was reduced to 0.53–0.58 m. 

Finally, when the Kalman filter and MAMD were implemented together, the average error decreased to 0.2–0.22 m. Inaccurate RSSIs are filtered in advance because the MAMD is fixed as 0.25 m, and the Kalman filter stabilized the calculation process.

#### 4.1.2. Location Tracking of Traveling Tag

**Figure 8:**
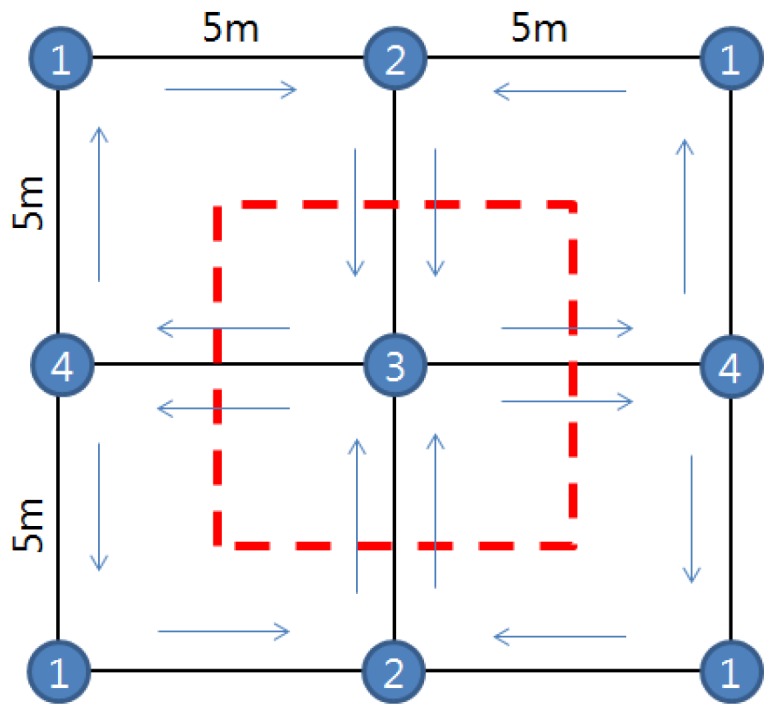
Scanning procedure and path of moving tag.

**Figure 9:**
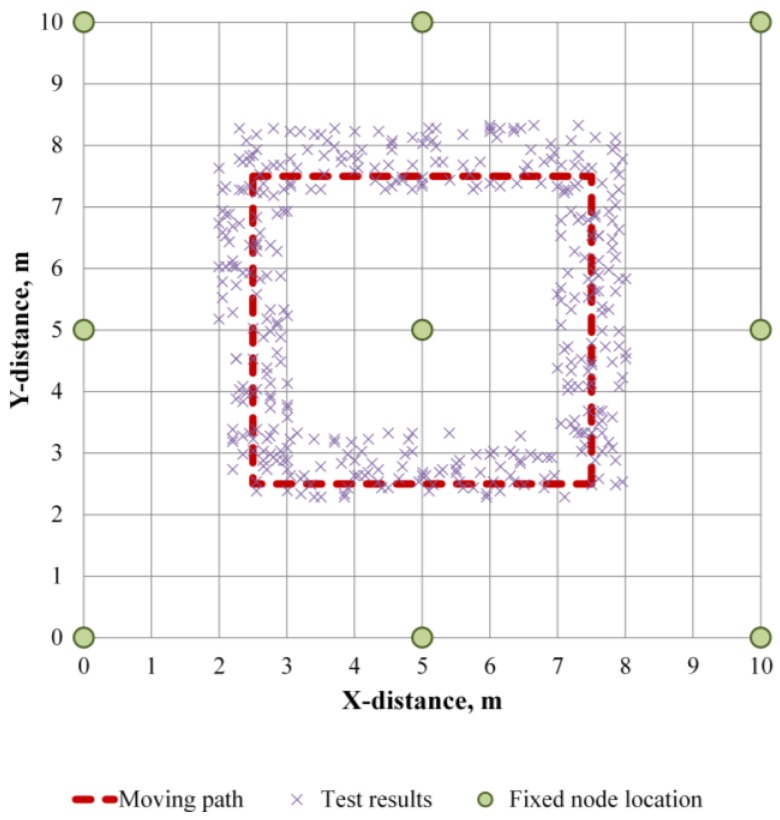
Tracking result of moving tag in underground construction site.

To evaluate the location tracking capability for a moving tag, eight nodes were used with the tag moving 10 times along a glide path in a 10 m × 10 m plane space. The arrangement of the fixed nodes and the scanning sequence are presented in Figure 8. Figure 9 shows the result. Table 3 shows the errors obtained from the moving tag. The average error of the moving tag was 0.10–0.47 m, which is slightly larger than the error of the fixed tag. This results from the influence of the error from the accelerometer. When the tag is moved in the X-axis direction, as shown by the data, it is consistently biased upwards relative to the path line since the test site is a slight gradient in the Y-axis direction. However, the result shows that the tag traveled along the predefined path. If an error range of 0.5 m is acceptable, the location tracking technique can be applied to the construction field. 

**Figure 10:**
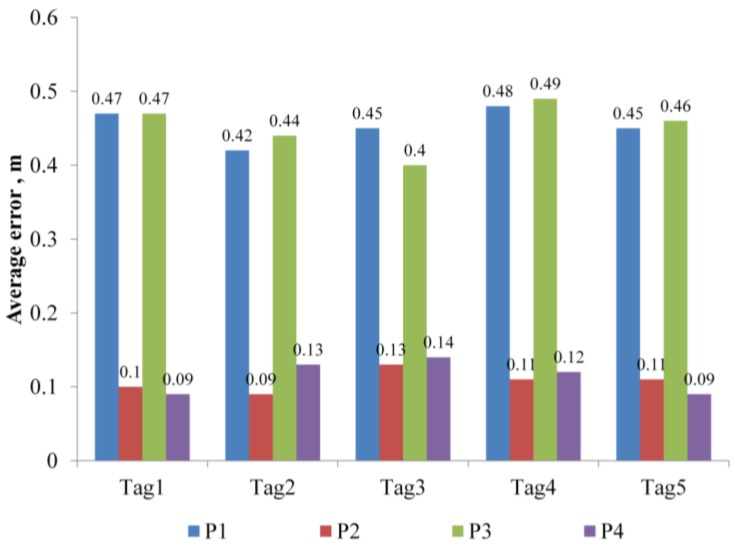
Average error evaluation of each tags.

Location tracking for five traveling nodes was also conducted. The nodes traveled along the predefined path in Figure 8 in the same direction. The results were assessed in terms of the difference between the tracked location data and the glide path. As shown in Figure 10, the error is not affected by the number of tags.

### 4.2. Evaluation of the RTL System for a Bridge Construction Site

**Figure 11:**
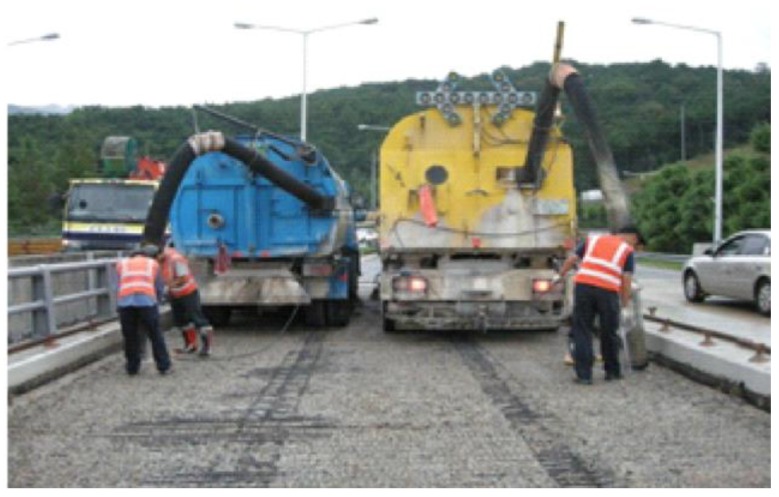
Bridge overlay site.

**Figure 12:**
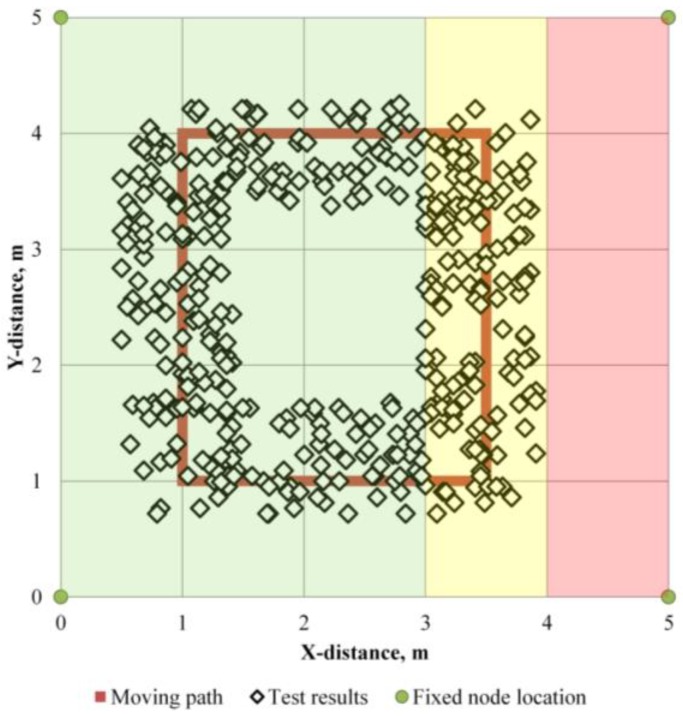
Tracking result of moving tag in a bridge overlay site.

A field test was conducted to evaluate the performance of the system at a bridge overlay site for an expressway. Figure 11 shows a picture of the site. Fixed nodes are placed at appropriate positions based on surveying, and a portable tag is horizontally attached to the helmet of a laborer. The field test is aimed at evaluating the technique in an open construction space. First, the location tracking of a moving laborer and an alert upon entering a red zone were checked. Second, the system was tested for recognizing that a laborer has fallen down on the ground for about 3 min while the laborers are moving and notifying the other laborers. To evaluate the location tracking, four nodes traveled along the glide path 10 times in a 5 m × 5 m plane space, and the location tracking capability was monitored. Figure 12 shows the result. When the tag is traveling, the average error is 0.32 m from the influence of the error induced by the accelerometer.

To investigate the error for the detection of entering the red zone, the glide path shown in Figure 12 was established, and the approach to the red zone out of the yellow zone was not observed. The result shows that the tag traveled along the predefined path. If the acceptable error range is 0.5 m, the location tracking technique can be used at a construction site. 

Location tracking of five laborers was conducted, and one of the helmets of the laborers was exposed to gravity horizontally for 3 min. The system recognizes the abnormality of the laborer and notifies the other laborers, and it takes about 2 min for each trial. This shows that the system could be used in the construction field. 

### 4.3. Discussion

For efficient laborer management and safety assurance, a real-time location system is implemented for a construction site in this study. Lee et al. [7] and Li et al. [6] addressed that the triangular survey method using an RSSI for location tracking of the movement of multiple laborers is limited in a confined construction field due to excessive errors involved. When the Kalman filter is introduced, the moving tag is barely identified around the fixed node.

To improve the both efficiency and accuracy, MAMD (maximum allowable movement distance) is implemented in the real-time location monitoring system. Moreover, an accelerometer is combined with a conventional sensor system. The MAMD proposed in this study is 0.25 m/s since the movement of laborers in a confined construction field such as a tunnel and underground space is relatively slower than usual. The optimal MAMD should be determined based on various field studies in the future.

Since this study was initiated, the wearable smart device market is rapidly growing. In the market, BLE devices combined with accelerometers are appearing. Thus, the realization of the suggested composition can be commercialized in the construction field as well.

Especially, an accelerometer can be utilized not only in location monitoring but also for the behavior of laborers based on the acceleration variation. In the future, various sensors also can be combined with the suggested system to monitor the health conditions and for safety monitoring of multiple laborers in the construction field.

## 5. Conclusions

To improve current RTL systems, an accelerometer was added, and an effective search algorithm based on a Kalman filter was suggested in this study. The system was implemented in a constrained tunnel and bridge construction site for the sustainable management and safety of laborers. The accelerometer with BLE can be used as an effective sensor for the detection of laborers’ movements. The maximum error was 3 m with triangular surveying and RSSI, which was reduced when the Kalman filter was incorporated. The MAMD can reduce the error and track multiple moving tags. When the sensor is fixed, the average error ranges 0.2–0.22 m, and when the sensor is moving, the average error ranges 0.1–0.47 m. The movement and location of laborers can be monitored with the system in a confined construction site. The safety and management of laborers can be monitored, and the characteristics of the laborers’ behavior can be observed in real time.

## Figures and Tables

**Table 1:** Comparison of wireless network techniques.

Contents	Wi-Fi	Bluetooth	ZigBee
Frequency Band, Ghz	2.4/5	2.4	868/915(MHz) 2.4
Max. transfer rate, Mbps	11–54	1	0.25
Max. transfer distance, m	100	10	10~75
Power Consumption, mW	800–1600	50/80	1/75
Network Configuration	P2P, Star	P2P, Star, Adhoc	P2P, Star, Mesh
Standardization	IEEE 802.11 Wi-Fi Alliance	IEEE 802.15.1 Bluetooth SIG	IEEE 802.15.4 ZigBee Alliance

**Table 2:** Results of error range for fixed tag.

Case	Average Error	Standard Deviation of Error	Maximum Error	Minimum Error	Number of Data
RSSI	1.56 m–2.82 m	0.59 m–1.22 m	2.92 m–4.88 m	0.30 m–0.70 m	1200
RSSI with Kalman filter	0.53 m–0.58 m	0.24 m–0.27 m	0.72 m–1.01 m	0.11 m–0.20 m	1200
RSSI with Kalman filter & MAMD	0.20 m–0.22 m	0.09 m–0.11 m	0.38 m–0.40 m	0.01 m–0.02 m	1200

**Table 3:** Results of error range for moving tag.

Path No.	Moving Direction	Ave. Error	Std. Error	Max. Error	Min. Error	No. of Data
P1	(2.5,2.5) → (2.5,7.5)	0.45	0.19	0.71	0.02	100
P2	(2.5,7.5) → (7.5,7.5)	0.10	0.21	0.47	0.01	100
P3	(7.5,7.5) → (7.5,2.5)	0.47	0.19	0.72	0.02	100
P4	(7.5,2.5) → (2.5,2.5)	0.11	0.20	0.48	0.00	100
